# Temporal Effect on PD‐L1 Detection and Novel Insights Into Its Clinical Implications in Non–Small Cell Lung Cancer

**DOI:** 10.1002/cam4.70262

**Published:** 2024-10-09

**Authors:** Gopal P. Pathak, Rashmi Shah, Mathieu Castonguay, Angela Cheng, John Fris, Rowan Murphy, Gail Darling, Alexander Ednie, Daniel French, Harry Henteleff, Aneil Mujoomdar, Madelaine Plourde, Alison Wallace, Zhaolin Xu

**Affiliations:** ^1^ Department of Pathology QEII Health Sciences Centre and Dalhousie University Halifax Nova Scotia Canada; ^2^ Division of Thoracic Surgery QEII Health Sciences Centre and Dalhousie University Halifax Nova Scotia Canada

**Keywords:** checkpoint inhibitor, driver mutation, immunotherapy, NSCLC, PD‐L1

## Abstract

**Objectives:**

Several studies rely on archived tissue blocks to assess the PD‐L1 scores; however, a detailed analysis of potential variations of scores between fresh and archived tissue blocks still lacks. In addition, the prognostic implications of PD‐L1 in lung cancers have not yet been completely understood. Here, we aimed to investigate the temporal variation in PD‐L1 scores from clinical samples and the clinical implications of PD‐L1 in non–small cell lung cancer (NSCLC).

**Methods:**

NSCLC cases from January 2005 to June 2023 were considered for this study, and PD‐L1 scores in archived and fresh tissue blocks were analyzed. Association of PD‐L1 with various driver mutations was explored, and implications of PD‐L1 in progression‐free survival (PFS) and overall survival (OS) were analyzed.

**Results:**

Our study revealed a significant disparity in PD‐L1 scores between archived and fresh tissue blocks, and a temporal variation in scores within 6 months of tissue acquisition. Advanced‐stage primary tumors, metastatic lymph nodes, and visceral pleural invasion revealed higher PD‐L1 expression as presented by tumor proportion score (TPS). Notably, in fully resected stage I/II NSCLC cases, OS was better in the high PD‐L1 (≥ 50% TPS) cohort with driver mutations compared to cases without driver mutations (hazard ratio—0.5129, 95% confidence interval 0.2058–1.084, *p* = 0.0779). In contrast, high PD‐L1 was associated with worse OS compared to no PD‐L1 (< 1% TPS) (hazard ratio—2.431, 95% confidence interval 1.144–6.656, *p* = 0.0242) in the cohort without driver mutations. Furthermore, the presence of a KRAS mutation favored the outcome of anti‐PD‐L1/PD1 immunotherapy in advanced NSCLC.

**Conclusion:**

PD‐L1 detection from tissue blocks was found to vary temporally, urging for a prioritized consideration for patients with marginal scores when archived blocks are employed for its detection. Prognostic roles of PD‐L1 were associated with driver mutations, and KRAS mutations favored the outcome of anti‐PD‐L1/PD1 therapy in advanced NSCLC.

## 1 Introduction

Interaction between programmed death‐ligand 1 (PD‐L1) and the inhibitory receptor programmed cell death protein 1 (PD1) on T cells plays a pivotal role in suppressing antitumor immune responses, thereby compromising immune activity against tumors. PD‐L1, a transmembrane protein primarily expressed by cancer cells and antigen‐presenting cells, initiates an immunosuppressive cascade upon binding with PD1 on T cells. PD‐L1 expression in the tumor microenvironment is often triggered by inflammatory cytokines, such as IFN‐γ, which are released by tumor‐infiltrating lymphocytes [1, 2]. Consequently, PD‐L1 expression may also indicate an immunosuppressive tumor microenvironment characterized by the presence of tumor‐infiltrating lymphocytes [3, 4]. Beyond its role in immune suppression, recent reports indicate that the intracellular domain of PD‐L1 is involved in various signaling functions related to cancer cell proliferation and metastasis. In vitro and mouse models have demonstrated that PD‐L1 collaborates with downstream partners to enhance cell proliferation and invasive properties by modulating the cytoskeleton [5, 6]. Remarkably, blocking PD‐L1 not only alleviates its immunosuppressive function but also inhibits the tumorigenic and metastatic potential of tumor cells [6].

Immune checkpoint inhibitor (ICI) therapy targeting the PD‐L1/PD1 axis has revolutionized the treatment of cancers, including non–small cell lung cancer (NSCLC) [7, 8, 9, 10, 11]. However, the field is still in its early stages, and despite the revolutionary strides in ICI therapy, applications in NSCLCs have shown only modest advantages in many cases, necessitating further insights into their optimal utilization [12, 13, 14]. The clinical assessment of PD‐L1 in tumor tissues represents the initial step in determining the potential for many immunotherapy applications. As such, PD‐L1 testing has become integral to modern NSCLC diagnostic practice, forming the basis for numerous clinical studies and treatment plans [15]. However, disparities in PD‐L1 scores, arising from various factors, can lead to potential deviations from expected outcomes. Such scores may not accurately reflect the true PD‐L1 status in tumors, thereby affecting overall cancer management [16, 17, 18]. In the absence of fresh tissues, many studies rely on archived samples to determine PD‐L1 status, but the potential deviation in PD‐L1 scores assessed from archived tumor tissues compared to fresh ones has not been fully explored [19, 20, 21]. In addition to PD‐L1 testing, molecular profiling of tumor tissues has become a standard practice to identify tumor characteristics and potential therapeutic targets. The presence of driver mutations and the expression of PD‐L1 have been found in various NSCLCs, yet the implications of their coexistence in the same tumors need to be further investigated to understand the therapeutic implications [22, 23, 24, 25, 26, 27]. Our study aims to compare PD‐L1 expression in archived and fresh tissue blocks, explore the relationship between PD‐L1 expression and driver mutations, and investigate the prognostic implications of PD‐L1 and driver mutations. The detailed analysis covers an array of NSCLC tumors and explores correlations of PD‐L1 in different tumor stages from patients receiving treatment at various time points.

## 2 Materials and Methods

### 2.1 Patients

Patients who underwent surgical resection or biopsy for NSCLC at the Queen Elizabeth II Health Sciences Centre (QEII HSC) in Halifax, Canada (from January 2005 to June 2023) and had PD‐L1 assessment performed were included in this retrospective study. All patients received treatment in accordance with national guidelines, adhering to standard care protocols. The study was approved by the Nova Scotia Health Authority's Research Ethics Board. Demographic and clinicopathological data, including patient age, gender, smoking history, cancer subtype, stage, vascular invasion, lymphatic invasion, and mutation status used in this analysis, were extracted from our in‐house database (QEII Lung Cancer Database and Tissue Bank), pathology reports, and medical records.

### 2.2 PD‐L1 and Molecular Analysis

PD‐L1 assessment was performed using anti‐PD‐L1 antibody clone 22C3 and an immunohistochemistry (IHC) assay platform (pharmDx assay; Dako). PD‐L1 expression was measured by Tumor Proportion Score (TPS), reported as the percentage of viable tumor cells with detectable partial or complete membranous PD‐L1 staining. Cases were defined as PD‐L1 positive when at least 1% of tumor cells stained for PD‐L1 (≥ 1% TPS); otherwise, they were deemed no PD‐L1 or PD‐L1 negative (< 1% TPS). PD‐L1 positive cases were divided into high PD‐L1 (≥ 50% TPS) or low PD‐L1 (1%–49% TPS) cases. PD‐L1 assessment started in 2017 at QEII HSC, and tissues were assessed regularly with an average collection‐to‐assessment time of approximately 1 month. Those samples were defined as fresh (non‐archived), and the PD‐L1 assessment was for diagnostic purposes. Tissues from 2016 and before were samples stored in the lung tumor archive and defined as archived samples. PD‐L1 assessment on archived samples was performed between 2017 and 2019 along with the regular diagnostic samples as part of the research study. For the archived samples, the average collection‐to‐assessment time was approximately 85 months. During the indicated period for PD‐L1 analysis, there were no significant changes in the assessment center that could potentially introduce any variation in test outcomes. Both fresh and archived samples represented the pretreatment diagnostic samples. Molecular analysis of the driver genes was queried from the in‐house database. The molecular analysis was performed using TruSight Tumor 15 and Illumina Focus Panel (Illumina). For the analysis of metastatic lymph nodes and pleural involvement, we relied on non‐matching metastatic lymph nodes due to the challenges associated with acquiring PD‐L1 and molecular data from both primary tumors and matched metastatic lymph nodes or pleural involvement in routine clinical procedures.

### 2.3 Statistical Analysis

GraphPad Prism 8 (GraphPad, San Diego, USA) was used for data analysis. Survival data was analyzed and plotted using the Kaplan–Meier method. Hazard ratio (HR) was computed using the log‐rank approach and was given at a 95% confidence interval (CI). PD‐L1 scores between the category groups were evaluated by chi‐squared or Fisher's exact test, as appropriate. All hypothesis tests were two‐sided, and a *p* value less than 0.05 was considered statistically significant. All early‐stage NSCLC patients included in the survival analysis underwent curative‐intent surgical resection, and none of the patients received adjuvant chemoimmunotherapy. For the early‐stage cohort, patients treated with targeted/ICI therapy at any point after disease progression or who died within 2 months of surgical intervention were excluded from the survival analysis. Cases from 2016 to 2019 were included in the survival analysis. Progression‐free survival was defined as the time after surgery until the point when recurrence or advancement of the disease was confirmed. To monitor the progression of early‐stage cases, follow‐up imaging after surgery was performed at an interval of 6 months for 2 years, and from the third year onward once every year up to 5 years when no progression was indicated. To study the implications of ICI on the outcome of advanced stage cases with respect to mutation status, patients who received at least five cycles of ICI therapy were considered for survival analysis from the ICI therapy cohort. This criterion was selected to exclude patients who did not tolerate ICI therapy due to adverse side effects and stopped the ICI treatment. For individual driver mutations in the survival analysis in the ICI category, we considered KRAS only as there were not sufficient cases with other driver mutations.

## 3 Results

### 3.1 PD‐L1 Detection in Archived and Fresh Tissue Blocks

**FIGURE 1:**
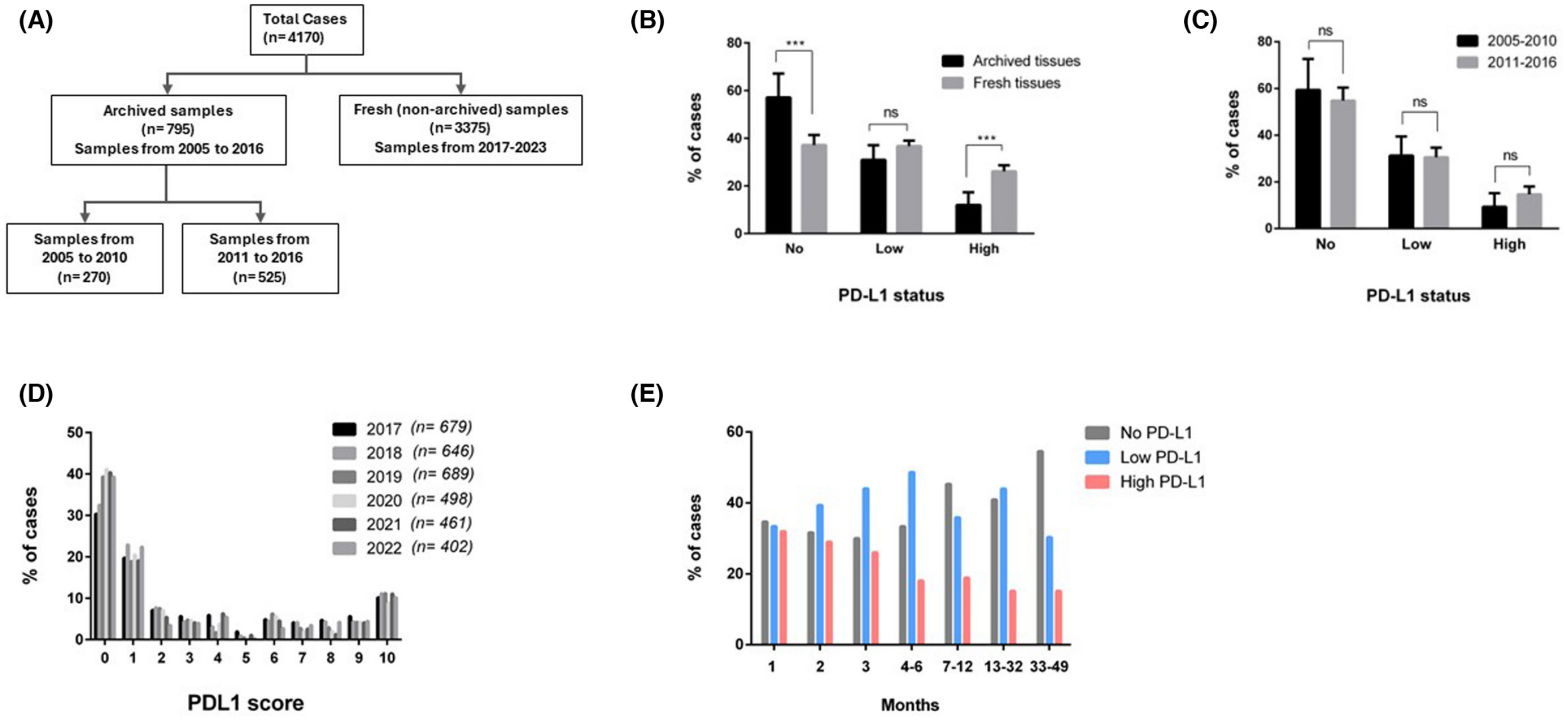
PD‐L1 detection in archived and fresh tissues. (A) Overview of tissue samples used for PD‐L1 analysis. (B) Annual PD‐L1 data from archived tissue blocks (2005–2016) and fresh tissue blocks (2017–2022), as outlined in (A), are plotted according to high (≥ 50% TPS), low (1%–49% TPS), and no PD‐L1 (< 1% TPS). (C) Comparison of annual PD‐L1 data between two archived groups [as outlined in (A)]. The data from 2005 to 2010 (average tissue acquisition to PD‐L1 assessment time of 122 months) is compared with the data from 2011 to 2016 (average tissue acquisition to assessment time of 48 months). *p* values are given as asterisks when significant or labeled as ns (not significant) in (B, C). (D) PD‐L1 TPS score (0–10) assigned for cases from 2017 to 2022 to demonstrate the trend in PD‐L1 expression (from fresh tissue blocks). Scores are defined as: 0 = no PD‐L1 expression, 1 = 1%–9% TPS, 2 = 10%–19% TPS, 3 = 20%–29% TPS, 4 = 30%–39% TPS, 5 = 40%–49% TPS, 6 = 50%–59% TPS, 7 = 60%–69% TPS, 8 = 70%–79% TPS, 9 = 80%–89% TPS, 10 = 90%–99% TPS, *n* refers to number of cases. (E) PD‐L1 status in tissue blocks assessed at the given time (in month) after the tissue acquisition (biopsy/surgery). Number of cases (*n*): 1 month = 842, 2 months = 610, 3 months = 100, 4–6 months = 72, 7–12 months = 53, 13–32 months = 62, and 33–49 months = 99.

We conducted a comprehensive analysis, categorizing cases into two groups, one based on archived tissue blocks and the other on fresh tissue blocks, to compare the PD‐L1 scores from those two groups (Figure 1A). The majority of cases were adenocarcinoma (about 74%) followed by squamous cell carcinoma (SCC) (about 18%) (Table 1). Our analysis revealed a significant difference in PD‐L1 status between archived and fresh tissue blocks. A higher proportion of PD‐L1 positive cases (63%) was evident in the fresh tissue cohort. In contrast, the archived tissue cohort had only about 45% of PD‐L1 positive cases (Figure 1B; Table 2). Importantly, among the archived tissue blocks, there was no significant change in PD‐L1 status between specimens collected from 2005 to 2010 and 2011 to 2016 (Figure 1C). Within fresh blocks, a consistent pattern of PD‐L1 scores existed without significant deviation (Figure 1D), indicating that significant variation existed between archived and fresh tissue blocks only. High PD‐L1 was observed in only about 13% of archived cases, compared to about 26% of fresh cases (Table 2).

**TABLE 1:** Patient characteristics and number of cases according to the histological types.

Characteristics	Number of cases (%)	Female (%)	Male (%)
All cases	4414 (100)	2487 (56.34)	1927 (43.66)
Histology types			
Adenocarcinoma	3245 (73.52)	1965 (61)	1280 (39)
Squamous cell carcinoma	793 (17.97)	340 (43)	453 (57)
Neuroendocrine	100 (2.26)	59 (59)	41 (41)
Sarcomatoid	55 (1.25)	23 (42)	32 (58)
Adenosquamous	23 (0.52)	18 (78)	5 (22)
Adenosquamous large cell	16 (0.36)	8 (50)	8 (50)
Large cell carcinoma	17 (0.38)	5 (29)	12 (71)
Others/unspecified	165 (3.74)	69 (42)	96 (58)

*Note:* Median age at diagnosis = 69 years (SD = 8.66).

**TABLE 2:** Histology type and PD‐L1 status.

Characteristics	Archived tissues	Fresh tissues	*p*
PD‐L1 in all cases (*n* = 4388)	*n* = 795	*n* = 3593	0.0001
TPS < 1%	438 (55.09)	1329 (36.99)	
TPS 1%–49%	252 (31.70)	1322 (36.79)	
TPS ≥ 50%	105 (13.21)	942 (26.22)	
Female cohort (*n* = 2475)	*n* = 419	*n* = 2056	0.0001
TPS < 1%	236 (56.32)	771 (37.50)	
TPS 1%–49%	129 (30.79)	740 (35.99)	
TPS ≥ 50%	54 (12.89)	545 (26.51)	
Male cohort (*n* = 1913)	*n* = 376	*n* = 1537	0.0001
TPS < 1%	202 (53.72)	558 (36.30)	
TPS 1%–49%	123 (32.71)	582 (37.87)	
TPS ≥ 50%	51 (13.56)	397 (25.83)	
Adenocarcinoma (*n* = 3226)	*n* = 544	*n* = 2682	0.0001
TPS < 1%	301 (55.33)	1028 (38.33)	
TPS 1%–49%	167 (30.70)	968 (36.09)	
TPS ≥ 50%	76 (13.97)	686 (25.58)	
SCC (*n* = 790)	*n* = 176	*n* = 614	0.0001
TPS < 1%	84 (47.73)	194 (31.60)	
TPS 1%–49%	69 (39.20)	264 (43.00)	
TPS ≥ 50%	23 (13.07)	156 (25.41)	
Neuroendocrine (*n* = 100)	*n* = 39	*n* = 61	0.71
TPS < 1%	31 (79.49)	47 (77.05)	
TPS 1%–49%	8 (20.51)	13 (21.31)	
TPS ≥ 50%	0	1 (1.64)	
Sarcomatoid (*n* = 55)	*n* = 24	*n* = 31	0.001
TPS < 1%	12 (50.00)	5 (16.13)	
TPS 1%–49%	6 (25.00)	3 (9.68)	
TPS ≥ 50%	6 (25.00)	23 (74.19)	
Other cases (*n* = 217)	*n* = 12	*n* = 205	0.0001
TPS < 1%	10 (83.33)	55 (26.83)	
TPS 1%–49%	2 (16.67)	74 (36.10)	
TPS ≥ 50%	0	76 (37.07)	

*Note:* Numbers in parentheses indicate percentages (%). Chi‐squared test was used to calculate the *p* values.

To further explore the variation over a shorter time interval, we analyzed PD‐L1 scores from blocks collected within a month and beyond, depending on sample availability. We found a decrease in the proportion of PD‐L1 positive cases even after a month of sample acquisition, with a shift towards no PD‐L1 from about 35% in 1‐month‐old tissue blocks to 56% in about 41‐month‐old blocks (Figure 1E). The proportion of high PD‐L1 cases decreased from about 32% in one‐month‐old tissue blocks to 15% in samples older than 22 months. Interestingly, the proportion of high PD‐L1 gradually diminished, with a significant decrease observed at 4–6 months (*p* = 0.013).

### 3.2 PD‐L1: Patient Gender and Histological Types

Subsequently, we aimed to investigate whether there was any correlation between PD‐L1 expression and gender. In archived tissue blocks, about 12.9% of the female cohort exhibited high PD‐L1, compared to 13.6% of the male cohort (Table 2). Among fresh tissue blocks, 26.5% of the female and 25.8% of the male cohort displayed high PD‐L1, while 37.5% of the female and 36.3% of the male cohort were PD‐L1 negative. PD‐L1 scores in archived tissue blocks were lower in most histological types than in fresh ones. An exception was the neuroendocrine cohort, in which PD‐L1 expression was generally low and no significant variation was observed between archived and fresh tissue blocks.

Analyzing adenocarcinoma, SCC, and sarcomatoid cohorts, we observed that the difference in PD‐L1 expression between female and male cases was less than 5% from fresh tissue blocks. Only among not‐otherwise‐specified cases, we found about 8% variation between genders (F > M, data not shown) in the high PD‐L1 group. Across major NSCLC histological types, there was no significant variation in PD‐L1 expression between female and male cases (Table S1).

### 3.3 PD‐L1 and Tumor Stage in NSCLC

**FIGURE 2:**
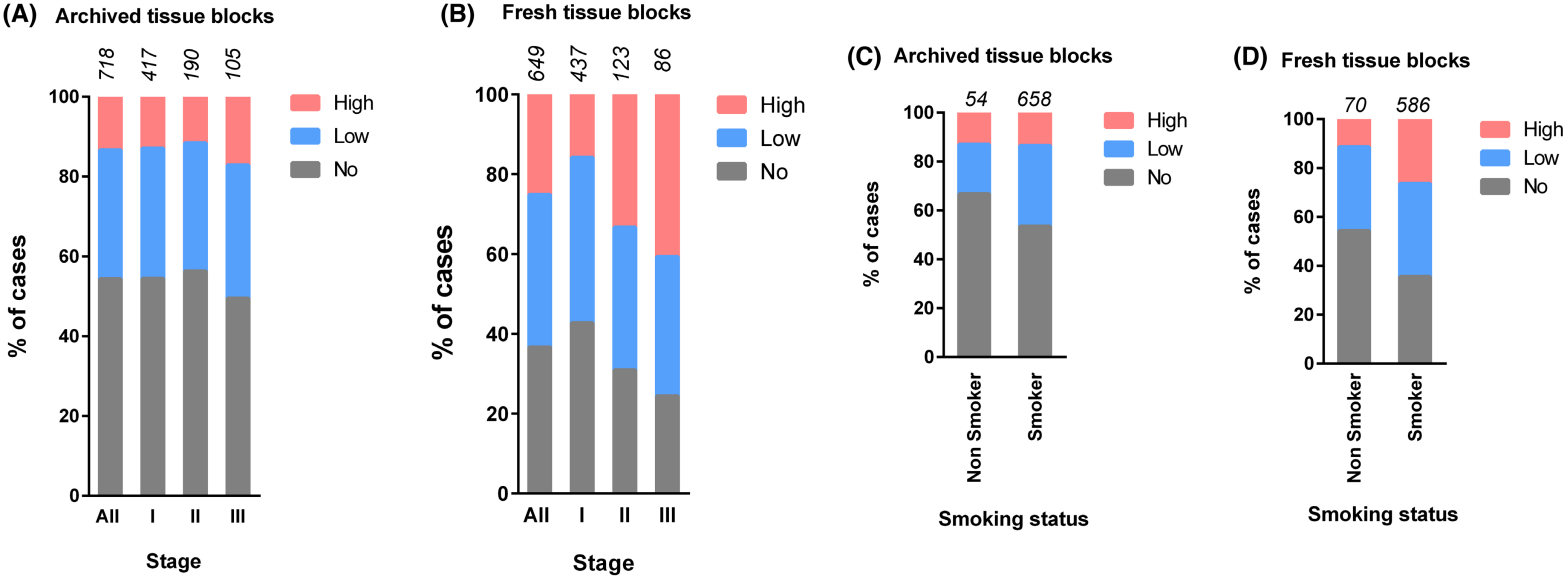
Tumor stage, smoking history, and PD‐L1 status. PD‐L1 expression in (A) archived tissues and (B) freshly acquired tissues is shown according to the stage. Smoking status and PD‐L1 scores are shown in (C) archived tissues and (D) freshly acquired tissues. PD‐L1 scores are shown as high (≥ 50% TPS), low (1%–49% TPS), and no PD‐L1 (< 1% TPS). The total number of cases (*n*) is shown on the top of the bar.

At the time of analysis, detailed information regarding tumor stage (anatomical staging) and patient history was available for 1483 cases enrolled in the tumor bank, with the majority belonging to stages I (*n* = 911) and II (*n* = 342). We investigated the relationship between tumor stage and PD‐L1 expression in archived and fresh tissue blocks. Stage I had lower PD‐L1 expression compared to stages II and III (Table 3). A significant increase in PD‐L1 expression in relation to the tumor stage was observed when scores from fresh tissue blocks were analyzed (Figure 2A; Table S3). Although the archived cases showed a similar trend of increasing PD‐L1 expression in advanced stages, the proportion of PD‐L1 positive cases was lower due to the variation in PD‐L1 detection between fresh and archived tissues (Figure 2B).

**TABLE 3:** Disease stage, smoking history, and PD‐L1 status.

Characteristics	Archived tissues	Fresh tissues	*p*
Stages (*n* = 1483)	*n* = 821	*n* = 662	
Stage I	464 (56.52)	447 (67.52)	0.0002
Stage II	218 (26.55)	124 (18.73)	
Stage III	131 (15.96)	88 (13.29)	
Stage IV	8 (0.97)	3 (0.45)	
PD‐L1 status			
All (stage I–IV, *n* = 1367)	*n* = 718	*n* = 649	
TPS < 1%	390 (54.32)	238 (36.67)	0.0001
TPS 1%–49%	232 (32.31)	248 (38.21)	
TPS ≥ 50%	96 (13.37)	163 (25.12)	
Stage I (*n* = 854)	*n* = 417	*n* = 437	
TPS < 1%	227 (54.44)	178 (42.69)	0.0002
TPS 1%–49%	136 (32.61)	173 (41.49)	
TPS ≥ 50%	54 (12.95)	86 (20.62)	
Stage II (*n* = 313)	*n* = 190	*n* = 123	
TPS < 1%	107 (56.32)	38 (30.89)	0.0001
TPS 1%–49%	61 (32.11)	44 (35.77)	
TPS ≥ 50%	22 (11.58)	41 (33.33)	
Stage III (*n* = 191)	*n* = 105	*n* = 86	
TPS < 1%	52 (49.52)	21 (24.42)	0.0002
TPS 1%–49%	35 (33.33)	30 (34.88)	
TPS ≥ 50%	18 (17.14)	35 (40.70)	
Never smoker (*n* = 124)	n = 54	*n* = 70	
TPS < 1%	36 (66.67)	38 (54.29)	0.23
TPS 1%–49%	11 (20.37)	24 (34.29)	
TPS ≥ 50%	7 (12.96)	8 (11.43)	
Past/current smoker (*n* = 1243)	*n* = 658	*n* = 586	
TPS < 1%	351 (53.42)	208 (35.49)	0.0001
TPS 1%–49%	217 (33.03)	223 (38.05)	
TPS ≥ 50%	89 (13.55)	155 (26.45)	

*Note:* Numbers in parentheses indicate percentages (%). Chi‐squared test was used to calculate the *p* values.

### 3.4 PD‐L1 and Smoking Status in NSCLC

Most patients in the tumor bank cohort were previous or current smokers, constituting about 91% of the study population. Approximately 45% of the non‐smoking cohort were PD‐L1 positive, compared to about 64% of patients with a smoking history when data from fresh blocks were analyzed (Figure 2C). Similarly, more cases with high PD‐L1 were found among patients with a smoking history compared to non‐smokers. The difference was particularly notable in fresh tissue blocks (Table 3; Table S1). Analyzing the archived blocks, 33% of the non‐smoking cohort were PD‐L1 positive compared to 46% of the smoking cohort (Table 3; Figure 2D). These results demonstrate that NSCLC patients with a smoking history have higher PD‐L1 expression compared to non‐smokers, emphasizing the potential influence of smoking history on PD‐L1 status.

### 3.5 Co‐Occurrence of PD‐L1 and Driver Mutations in Primary Tumors

**FIGURE 3:**
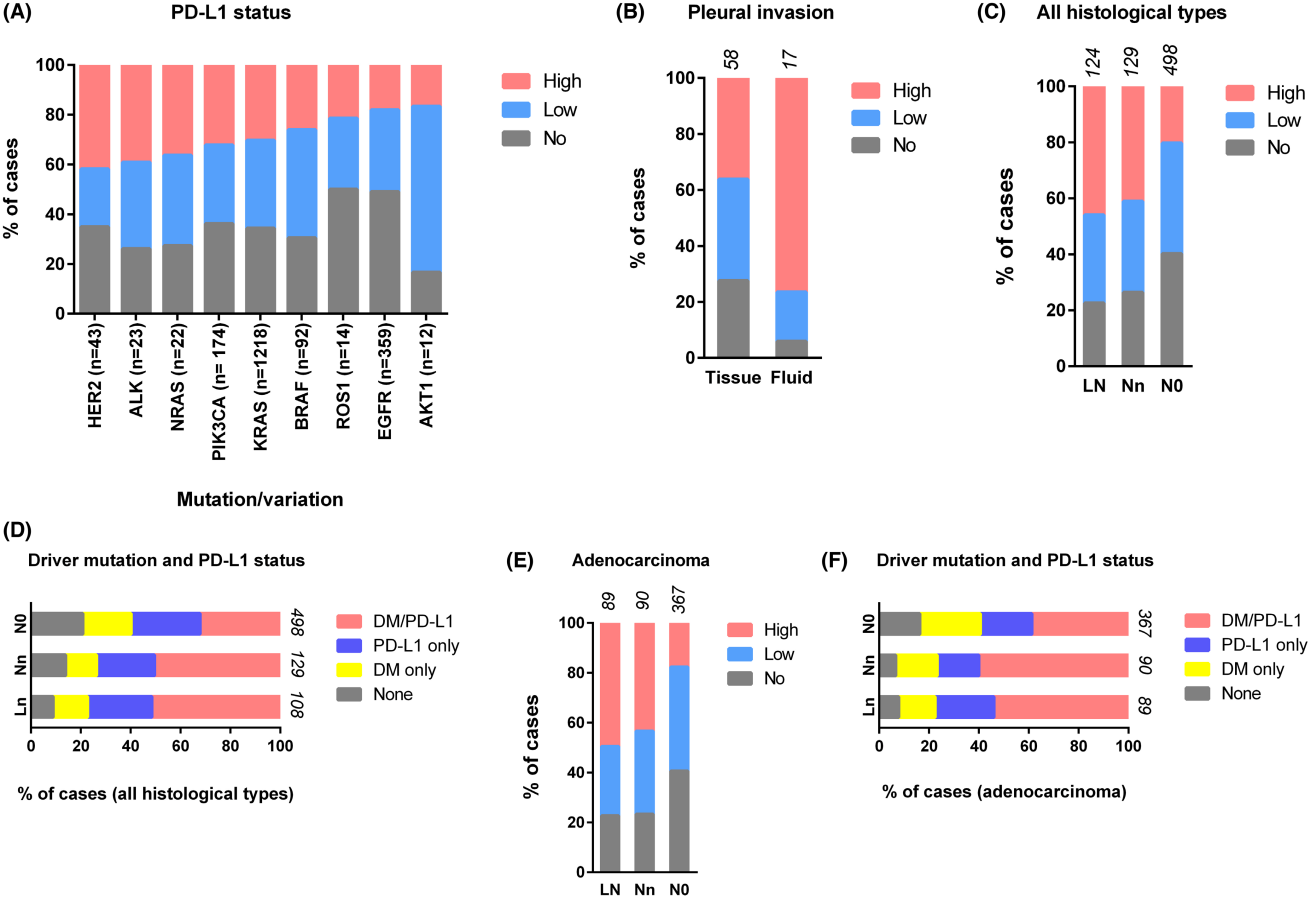
PD‐L1 expression and driver mutations. (A) Co‐existence of PD‐L1 expression and driver mutations in primary lung tumors. (B) PD‐L1 expression in pleural invasion. (C) PD‐L1 expression and (D) driver mutations and PD‐L1 expression (≥ 1% TPS) in metastatic lymph nodes (LN), and primary tumors with (Nn) or without (N0) lymph node involvement in all tissue types. (E) PD‐L1 expression and (F) driver mutations and PD‐L1 expression (≥ 1% TPS) in metastatic lymph nodes (LN), and primary tumors with (Nn) or without (N0) lymph node involvement in adenocarcinoma cases. PD‐L1 scores are given as high (≥ 50% TPS), low (1%–49% TPS), and no PD‐L1 (< 1% TPS) expression in (A–C, E). The total number of cases (*n*) is shown on the bar.

PD‐L1 expression was observed to coexist with various driver mutations, with molecular analysis predominantly conducted in adenocarcinoma and other selected cases following clinical diagnostic protocols. We investigated 1957 all‐histological type and 1830 adenocarcinoma cases with a driver mutation/alteration from fresh tissue blocks. Positive PD‐L1 expressions for each of these driver mutations ranged from 50% to 83%. More than 60% of cases with KRAS, PIK3CA, HER2, NRAS, AKT1, and ALK1 mutations were PD‐L1 positive (Table 4). Conversely, the proportion of PD‐L1 positive cases was low in the EGFR and ROS1 positive cohort. Additionally, the EGFR and ROS1‐positive cohort showed relatively fewer cases with high PD‐L1, while the HER2, ALK, NRAS, PIK3CA, and KRAS‐positive cohort exhibited more cases with high PD‐L1 (Figure 3A).

**TABLE 4:** Driver mutations and PD‐L1 expression.

Characteristic	KRAS	EGFR	PIK3CA	BRAF	HER2	NRAS	ROS1	AKT1	ALK1
All histological types									
TPS < 1%	418 (34.32)	176 (49.03)	63 (36.21)	28 (30.43)	15 (34.9)	6 (27.27)	7 (50)	2 (16.67)	6 (26.09)
TPS 1%–49%	431 (35.19)	118 (32.87)	55 (31.61)	40 (43.48)	10 (23.3)	8 (36.36)	4 (28.57)	8 (66.67)	8 (34.78)
TPS ≥ 50%	369 (30.30)	65 (18.11)	56 (32.18)	24 (26.09)	18 (41.9)	8 (36.36)	3 (21.43)	2 (16.67)	9 (39.13)
Total cases	1218	359	174	92	43	22	14	12	23
Adenocarcinoma cases									
TPS < 1%	410 (35.47)	175 (49.86)	63 (36.21)	26 (29.89)	14 (35)	6 (28.6)	6 (46.15)	1 (16.7)	4 (21.05)
TPS 1%–49%	412 (35.64)	111 (31.62)	55 (31.61)	37 (42.53)	10 (25)	7 (33.3)	4 (30.77)	4 (66.7)	7 (36.84)
TPS ≥ 50%	334 (28.89)	65 (18.52)	56 (32.18)	24 (27.59)	16 (40)	8 (38.1)	3 (23.08)	1 (16.7)	9 (39.13)
Total cases	1156	351	137	87	40	21	13	6	19

*Note:* Data from fresh tissue blocks (2017–2022). Numbers in parentheses indicate percentages (%).

### 3.6 PD‐L1 and Driver Mutations in Pleural Involvement

We analyzed 75 cases with pleural involvement, of which 58 cases were the pleural tissues with cancer involvement and 17 cases were malignant pleural fluid. In pleural tissues with cancer involvement, about 72% (42/58) of cases demonstrated positive PD‐L1 expression, with 36% (21/58) of cases showing high PD‐L1 (Figure 3B). Interestingly, in malignant pleural fluid, about 94% (16/17) of cases demonstrated positive PD‐L1 expression, with 76% (13/17) of cases displaying high PD‐L1 (Figure 3B). Driver mutation and PD‐L1 status were available for 55 pleural tissues and 14 malignant pleural fluid cases. Approximately 57% (8/14) of malignant pleural fluid cases had both positive PD‐L1 expression and a driver mutation, while only one case had negative PD‐L1 expression but a driver mutation (Figure S1A). In pleural tissues with cancer involvement, 49% (27/55) of cases exhibited both positive PD‐L1 expression and a driver mutation, about 24% (13/55) of cases had positive PD‐L1 expression but no driver mutation, about 14% (8/55) of cases had a driver mutation only (without PD‐L1 expression), and the remaining cases (7/55) had neither PD‐L1 expression nor a driver mutation (Figure S1B).

### 3.7 PD‐L1 and Driver Mutations in Primary Tumors With and Without Lymph Node Involvement

We conducted an analysis of primary tumors with or without lymph node involvement to investigate the status of PD‐L1 in tumors with local progression. Interestingly, we found that more than 73% (95/129) of cases with local lymph node involvement had positive PD‐L1 expression in the primary tumors, compared to only about 60% (298/498) of tumors without lymph node involvement (Figure 3C; Table S3). Among them, about 41% (53/129) of primary tumors with and only about 20% (101/498) of primary tumors without lymph node involvement demonstrated high PD‐L1. About 14% (18/129) of primary tumors with lymph node involvement had neither a driver mutation nor PD‐L1 expression, whereas about 21% (104/498) of tumors without lymph node involvement fell into this category (Figure 3D). Approximately 50% (65/129) of primary tumors with lymph node involvement and only about 32% (160/498) of primary tumors without lymph node involvement had both PD‐L1 expression and driver mutations.

### 3.8 PD‐L1 and Driver Mutations in Metastatic Lymph Nodes

Next, we analyzed metastatic lymph nodes originating from NSCLC to study the PD‐L1 status in such cases. Approximately 77% (96/124) of metastatic lymph nodes were PD‐L1 positive (Figure 3C; Table S3). Remarkably, about 46% (57/124) of cases demonstrated high PD‐L1, underscoring the importance of PD‐L1 in progression to lymph nodes. In metastatic lymph nodes, about 13% (14/108) of PD‐L1 negative cases had driver mutations, and only about 8% (9/108) of cases lacked both a driver mutation and PD‐L1 expression (Figure 3D; Table S3). Remarkably, approximately 51% (55/108) of metastatic lymph nodes had both positive PD‐L1 and a driver mutation (Figure 3D). Similar trends in PD‐L1 expression and the presence of driver mutations were observed in the adenocarcinoma cohort (Figure 3E,F; Table S3). These data demonstrate a comparable PD‐L1 status in metastatic lymph nodes and primary tumors with lymph node involvement, reflecting the implications of PD‐L1 in cancer progression and invasion.

### 3.9 High PD‐L1 and Driver Mutations Influence Survival Outcome in Early‐Stage NSCLC

**FIGURE 4:**
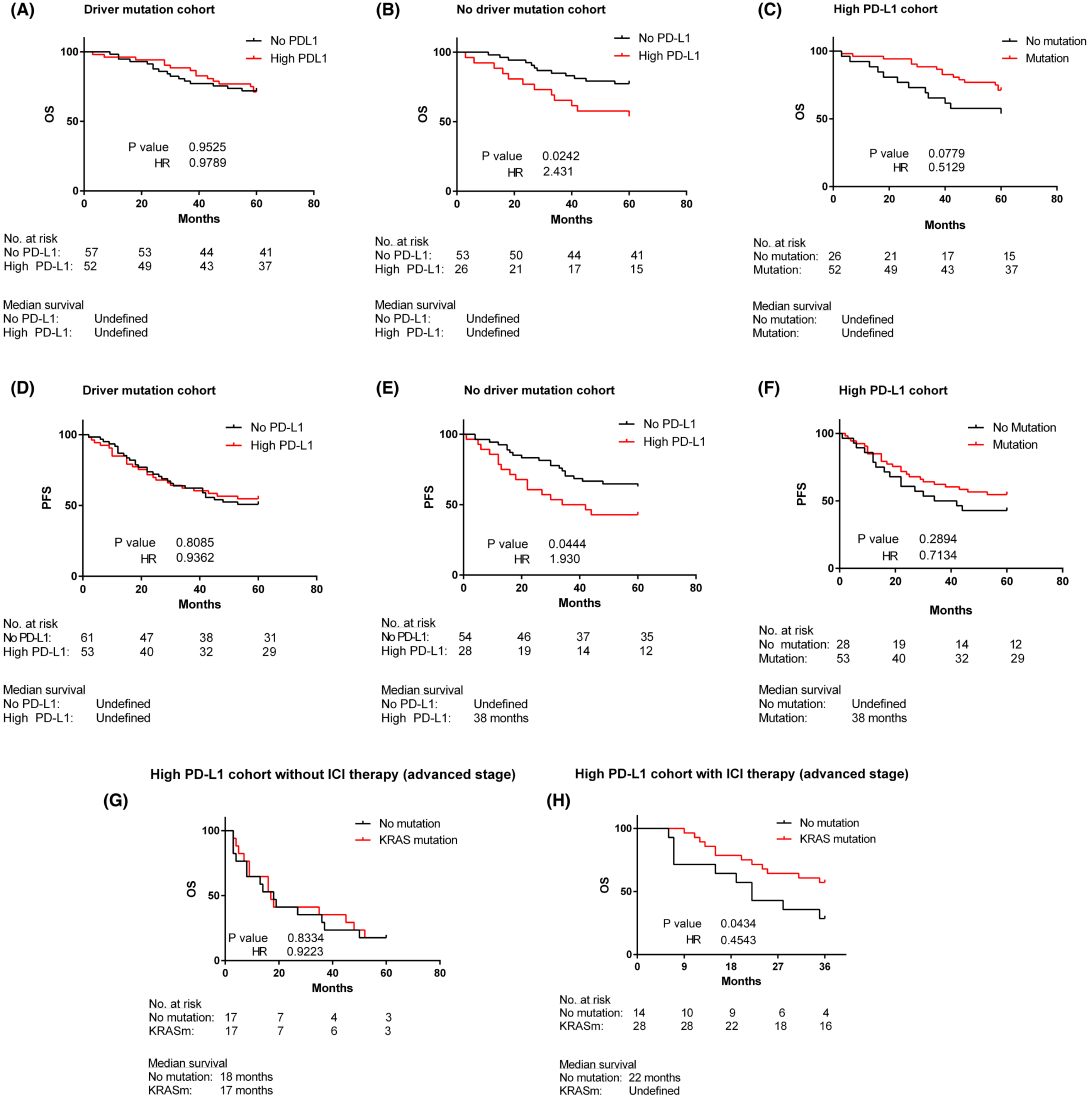
PD‐L1 and survival outcomes in adenocarcinoma (NSCLC). (A, B) Five‐year overall survival in relation to PD‐L1 status and presence (A) or (B) absence of driver mutations in stage I/II adenocarcinoma. (C) Five‐year overall survival in high PD‐L1 cohort with or without driver mutations in stage I/II adenocarcinoma. (D– F) Progression‐free survival as in (A– C). Patients did not receive any targeted or ICI therapy. (G) Five‐year overall survival of advanced stage adenocarcinoma patients with high PD‐L1 and KRAS mutations compared to the high PD‐L1 cohort without driver mutations. Patients did not receive any ICI therapy. (H) Three‐year overall survival in advanced stage adenocarcinoma (high PD‐L1 cases) with a KRAS mutation or without driver mutations following anti‐PD‐L1/PD1 treatment (KRASm: KRAS mutation).

We investigated cases after 2016, specifically focusing on the effect of driver mutations and PD‐L1 on survival outcome among stage I/II adenocarcinoma patients. All patients underwent curative‐intent surgery, and none of the patients received adjuvant immunotherapy. In the driver mutation cohort, PD‐L1 expression did not have a significant effect on overall survival (OS) (Figure 4A). Intriguingly, in the cohort without driver mutations, high PD‐L1 was associated with a significantly worse outcome compared to no PD‐L1 (HR 2.431, 95% CI 1.144–6.656, *p* = 0.0242) (Figure 4B). We analyzed the high PD‐L1 cases to confirm that driver mutations influence survival outcome in this cohort. Among the high PD‐L1 cases, the driver mutation cohort had a better survival outcome than the no driver mutation cohort (HR 0.5129, 95% CI 0.2058–1.084, *p* = 0.0779) (Figure 4C).

Progression‐free survival (PFS) analysis showed a similar trend to OS. Among cases with driver mutations, there was no significant difference in PFS between high PD‐L1 and no PD‐L1 (Figure 4D). However, in the no driver mutation cohort, PFS was significantly lower for the high PD‐L1 compared to no PD‐L1 cases (HR 1.930, 95% CI 1.024–4.302, *p* = 0.0444) (Figure 4E). The median PFS for the high PD‐L1 cases in the no driver mutation cohort was 38 months, whereas for the PD‐L1 negative cases, it could not be defined in the given period (Figure 4F). Among PD‐L1 negative cases, driver mutations favored a negative outcome (OS—HR 1.291, 95% CI 0.6151–2.707, *p* = 0.5013) and (PFS—HR 1.469, 95% CI 0.8434–2.556, *p* = 0.1773) (Figure S2A,B) over no driver mutation. These results indicate that PD‐L1 and driver mutations influence the survival outcome.

The KRAS mutation was the predominant driver mutation in our study population, along with a smaller number of other mutations/variations. Therefore, we analyzed the KRAS mutant‐only cases and confirmed that the KRAS mutation showed a similar outcome compared to cases with no driver mutation (Figure S2C,D).

### 3.10 KRAS Mutations and Outcome of ICI Therapy in Advanced Stage NSCLCs

In advanced‐stage adenocarcinoma with high PD‐L1 and no ICI intervention, there was no remarkable difference in survival outcome with respect to a KRAS mutation. The cohort with KRAS mutation had a median survival of 17 months compared to 18 months for those without driver mutations after the diagnosis. The difference was not statistically significant (HR = 0.9223, 95% CI: 0.4339–1.936, *p* = 0.8334) (Figure 4G). Conversely, with ICI therapy, the outcome was better in the KRAS mutation cohort compared to the no driver mutation cohort in advanced stage adenocarcinoma with high PD‐L1 (HR = 0.4543, 95% CI: 0.1538–1.000, *p* = 0.0434) (Figure 4H). The median survival after the start of ICI therapy in the cohort without driver mutations was 22 months, while for the KRAS mutation cohort, it could not be defined within the given period.

## 4 Discussion

Due to the lack of fresh tissue blocks, several studies have relied on archived tumor tissue blocks for PD‐L1 assessment, and this analysis has been applied in several clinical trials [20, 23]. Limited information is available about the effect on PD‐L1 scores when archived tissues are used for PD‐L1 analysis in clinical practices. To address this issue, we conducted a comparative analysis, assessing PD‐L1 status in both archived and fresh tissue blocks in our repository. Our findings revealed diminished PD‐L1 expression in archived tissue blocks, emphasizing the importance of conducting PD‐L1 testing on fresh tissue blocks, preferably within a month of acquisition. The diminished PD‐L1 detection can be attributed to the unstable nature of the extracellular domain of PD‐L1 over time, leading to the loss of signal in older tissue blocks [28]. Studies have shown that the structural integrity of PD‐L1 epitopes recognized by various antibodies (including clone 22C3) can be affected by humidity and temperature in stored FFPE tissues, causing a reduction in the immunoreactivity of anti‐PD‐L1 antibodies, leading to a significant signal loss [29]. These observations highlight the importance of considering appropriate storage measures for FFPE blocks to preserve epitope integrity and ensure accurate immunohistochemical analysis. The validity and relevance of these findings are underscored by their grounding in authentic “real‐world” clinical practices. The imperative for meticulous normalization measures becomes pronounced, particularly in cases where archived blocks are employed, urging a prioritized consideration for patients with marginal PD‐L1 scores. Moreover, it is strongly advocated that reporting practices evolve to incorporate the temporal interval between tissue acquisition and PD‐L1 assessment, thereby enhancing the precision and reliability of the data.

A higher PD‐L1 expression was associated with specific mutations (HER2, ALK, NRAS, PIK3CA, and KRAS). EGFR mutation was associated with lower PD‐L1 expression, as seen in our previous study [30]. Additionally, we had reported a correlation of the frequency of mutations with cancer type and patient characteristics [27]. The current study involves a detailed analysis of a larger cohort (archived and fresh tissue blocks) offering advanced insights into PD‐L1 expression, driver mutations, and their clinical implications. We observed that a high PD‐L1 status correlated with tumor stage and smoking status, consistent with previous findings in the literature [31]. Notably, pleural invasion and metastatic lymph nodes were associated with significantly higher PD‐L1 expression. Interestingly, primary tumors with lymph node involvement demonstrated a larger proportion of cases with high PD‐L1 expression compared to those without lymph node involvement, and the pattern in PD‐L1 expression and driver mutations in primary tumors with lymph node involvement align to that of metastatic lymph nodes. The high PD‐L1 in such instances may reflect an immunosuppressive feature of tumor cells facilitating nodal metastasis and pleural invasion [24, 32, 33].

Our study highlights the role of driver mutations and high PD‐L1 status on survival outcomes in early‐stage NSCLC patients without distant metastasis. In early‐stage cases with curative intent surgery, high PD‐L1 was associated with a worse outcome in cohorts without driver mutations. Conversely, cases with high PD‐L1 and driver mutations demonstrated better survival outcomes than those without driver mutations. The data is based on the analysis of the early‐stage cases that did not receive any targeted/ICI therapy at any point. This study helps to explain the conflicting outcomes reported previously, as some reports suggested PD‐L1 played no prognostic roles [34, 35], while others indicated its association with worse prognosis [36, 37] or better outcomes [38].

Understanding the prognostic implications of PD‐L1 in early‐stage NSCLC is crucial for developing effective therapeutic approaches. Early‐stage primary tumors can serve as a source of neoantigens for priming and expansion of tumor‐specific lymphocytes, enhancing their antitumor response [39]. Higher PD‐L1 expression is associated with abundant tumor‐infiltrating lymphocytes, especially in tumors with KRAS mutations [40]. Cases with high PD‐L1 expression may experience an increased antitumor response once the inhibitory effect is blocked or the tumor is removed, potentially leading to a better outcome. The benefits of neoadjuvant chemoimmunotherapy in resectable cases further highlight the importance of the PD‐L1 axis in early‐stage NSCLC management [41, 42]. Our data offer preliminary insights into the role of driver mutations and PD‐L1 for risk group determination and urge further investigations to shed light on such implications. Moreover, such information can also be important for personalized vaccine development based on the patient's tumor molecular profile if these outcomes are associated with neoantigen presentation and immune response. In pancreatic ductal adenocarcinoma, tumor neoantigens with specific mutations have been reported to be associated with tumor‐free survival in resected patients [43]. Potential applications of these neoantigens for personalized vaccine development have been recently demonstrated [43, 44]. Interestingly, epitopes from driver mutations such as KRAS and PIK3CA were also among the predicted neoantigens [44]. Furthermore, driver mutations are also associated with a high tumor mutation burden, and the latter can contribute to antigen presentation, immune infiltration, and a better outcome in certain cases [45, 46, 47, 48].

In advanced stage cases, ICI therapy demonstrated improved patient outcomes, particularly in those with KRAS mutations. It has been reported that the mutational landscape influences response to ICIs in advanced stage NSCLCs, and patients with high PD‐L1 and select driver mutations had the best response to anti‐PD‐L1/PD1 therapy [49, 50, 51, 52]. These reports mostly reflect the outcomes in advanced stage NSCLCs, which are generally consistent with our findings. Our data reflect a retrospective analysis from a single center, with no significant changes in diagnostic and treatment practices during the study period. Our report offers a novel insight into the practical consideration and clinical implications of PD‐L1, which can be helpful for the therapeutic advancement and better management of NSCLC.

## Author Contributions

**Gopal P. Pathak:** conceptualization (equal), data curation (equal), formal analysis (lead), investigation (equal), methodology (equal), writing – original draft (lead), writing – review and editing (equal). **Rashmi Shah:** data curation (supporting), formal analysis (supporting), investigation (supporting), writing – review and editing (supporting). **Mathieu Castonguay:** data curation (supporting), investigation (supporting), writing – review and editing (supporting). **Angela Cheng:** data curation (supporting), writing – review and editing (supporting). **John Fris:** data curation (supporting), project administration (supporting), writing – review and editing (supporting). **Rowan Murphy:** resources (supporting), writing – review and editing (supporting). **Gail Darling:** resources (supporting), writing – review and editing (supporting). **Alexander Ednie:** resources (supporting), writing – review and editing (supporting). **Daniel French:** resources (supporting), writing – review and editing (supporting). **Harry Henteleff:** resources (supporting), writing – review and editing (supporting). **Aneil Mujoomdar:** resources (supporting), writing – review and editing (supporting). **Madelaine Plourde:** resources (supporting), writing – review and editing (supporting). **Alison Wallace:** resources (supporting), writing – review and editing (supporting). **Zhaolin Xu:** conceptualization (equal), data curation (lead), formal analysis (equal), funding acquisition (lead), investigation (lead), resources (lead), supervision (lead), writing – original draft (equal), writing – review and editing (equal).

## Ethics Statement

The study was approved by the Nova Scotia Health Authority's Research Ethics Board. A written informed consent was obtained from some participants in the study and a waiver/exempt of consent was granted by the REB/Ethics Committee for other participants (REB file #1013243).

## Conflicts of Interest

The authors declare no conflicts of interest.

## Supporting information


**Figure S1.** Status of PD‐L1 expression (≥ 1% TPS) and driver mutations in malignant pleural fluid (A) and pleural tissues with cancer involvement (B).
**Figure S2.** (A) Five‐year overall survival and (B) progression‐free survival in no PD‐L1 cohort with or without driver gene mutations. (C) Five‐year overall survival and (D) progression‐free survival in the high PD‐L1 cohort with a KRAS mutation or without driver gene mutations.

**Table S1.** Relationship of gender status and smoking history on PD‐L1 expression (PD‐L1 assessed from fresh tissues).
**Table S2.** PD‐L1 expression and disease status in NSCLC cases from the tumor bank cohort (PDL1 assessed from fresh tissues).
**Table S3.** PD‐L1 and driver gene mutation in metastatic lymph nodes and primary tumors with and without lymph node involvement.

## Data Availability

Upon request, non‐confidential information may be available from the corresponding author.
