# Supplementary figures and images for: Temporal Effect on PD‐L1 Detection and Novel Insights Into Its Clinical Implications in Non–Small Cell Lung Cancer
Source: Cancer Med. 2024 Oct 9;13(19):e70262. doi: 10.1002/cam4.70262 (PMC11462595; doi:10.1002/cam4.70262)

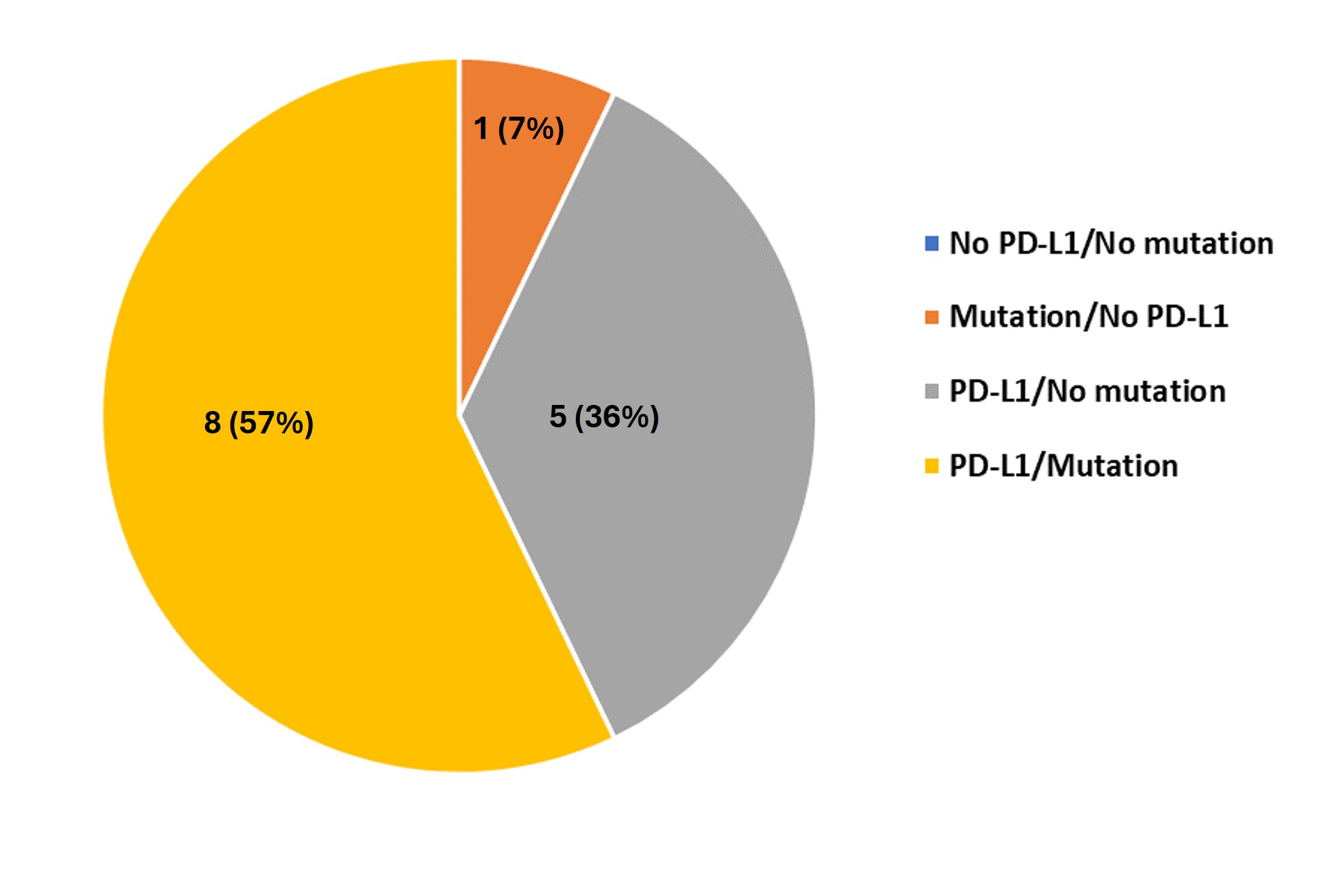

Supplement: Supplementary file 1 — Figure S1. Status of PD‐L1 expression (≥ 1% TPS) and driver mutations in malignant pleural fluid (A) and pleural tissues with cancer involvement (B). Figure S2. (A) Five‐year overall survival and (B) progression‐free survival in no PD‐L1 cohort with or without driver gene mutations. (C) Five‐year overall survival and (D) progression‐free survival in the high PD‐L1 cohort with a KRAS mutation or without driver gene mutations. [file CAM4-13-e70262-s002.zip › Figure S1A.jpg]

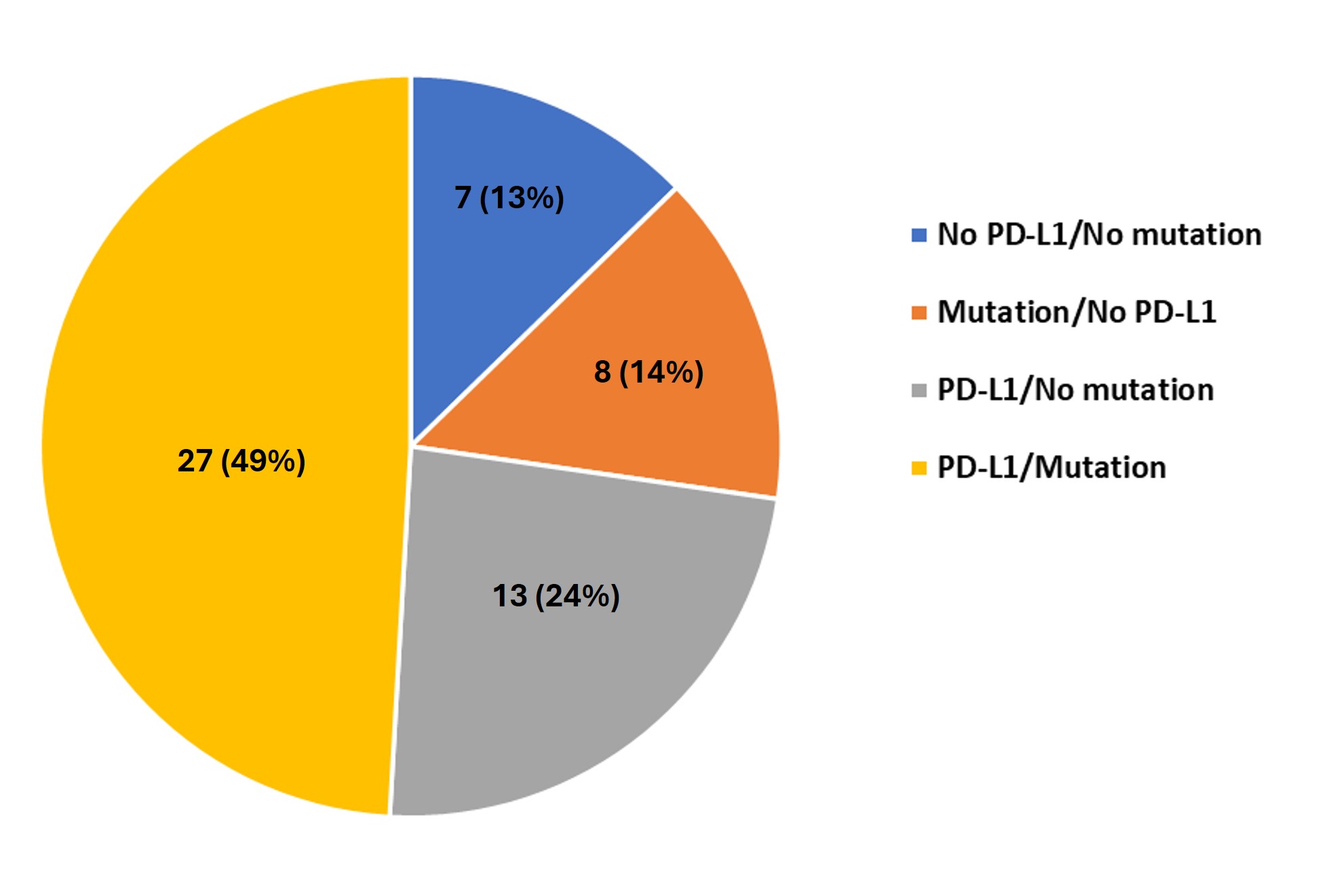

Supplement: Supplementary file 1 — Figure S1. Status of PD‐L1 expression (≥ 1% TPS) and driver mutations in malignant pleural fluid (A) and pleural tissues with cancer involvement (B). Figure S2. (A) Five‐year overall survival and (B) progression‐free survival in no PD‐L1 cohort with or without driver gene mutations. (C) Five‐year overall survival and (D) progression‐free survival in the high PD‐L1 cohort with a KRAS mutation or without driver gene mutations. [file CAM4-13-e70262-s002.zip › Figure S1B.jpg]

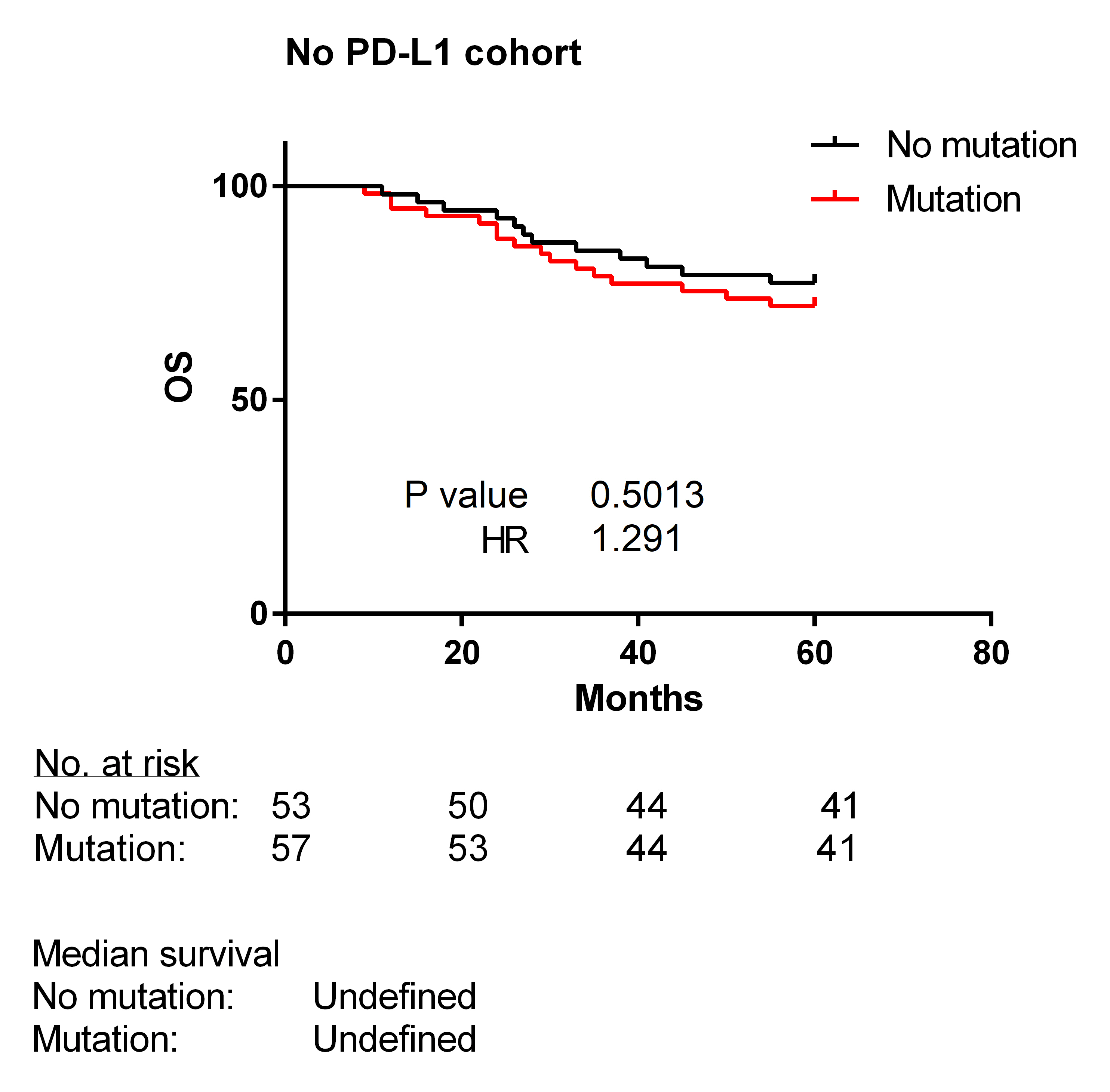

Supplement: Supplementary file 1 — Figure S1. Status of PD‐L1 expression (≥ 1% TPS) and driver mutations in malignant pleural fluid (A) and pleural tissues with cancer involvement (B). Figure S2. (A) Five‐year overall survival and (B) progression‐free survival in no PD‐L1 cohort with or without driver gene mutations. (C) Five‐year overall survival and (D) progression‐free survival in the high PD‐L1 cohort with a KRAS mutation or without driver gene mutations. [file CAM4-13-e70262-s002.zip › Figure S2A.jpg]

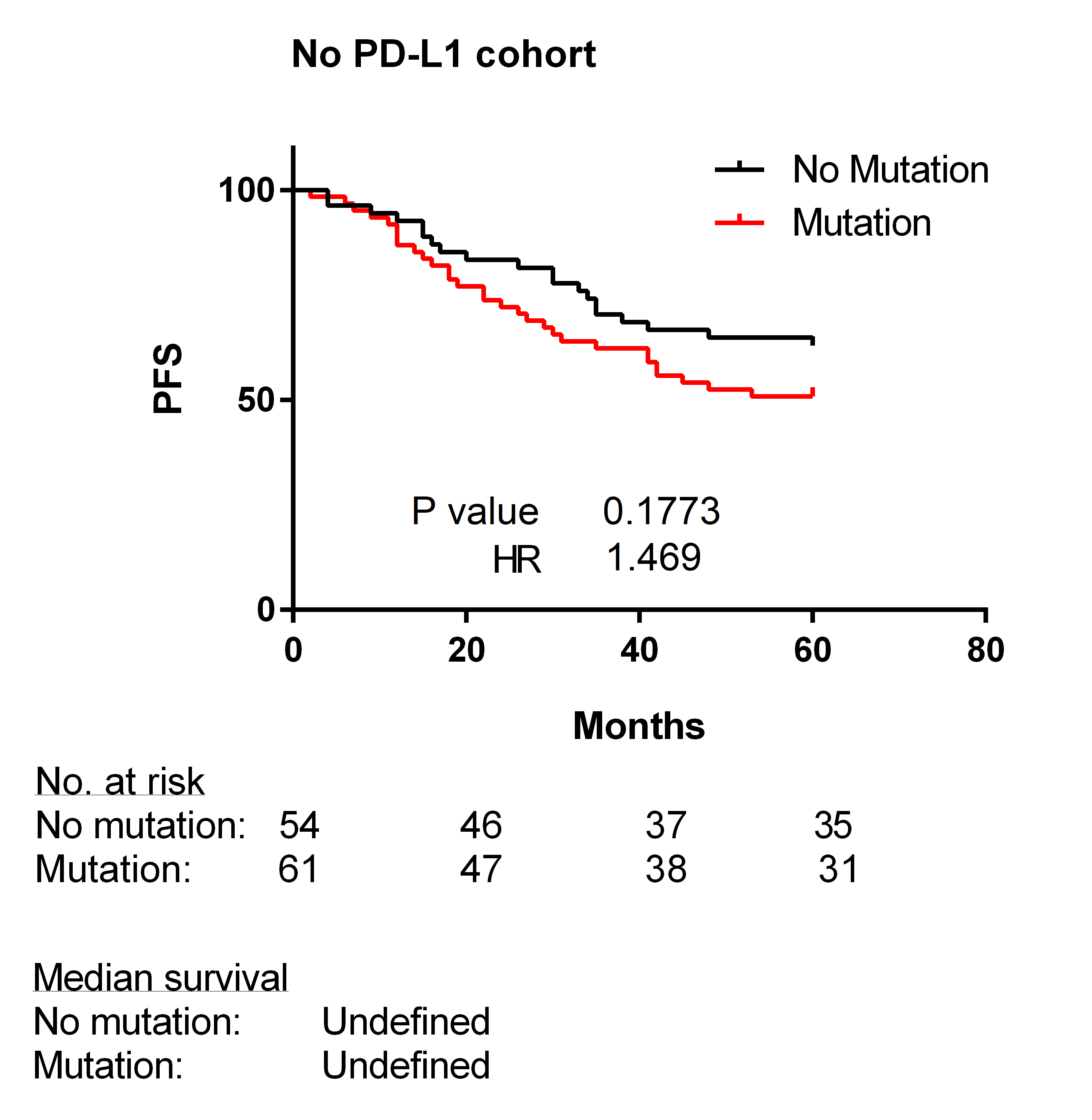

Supplement: Supplementary file 1 — Figure S1. Status of PD‐L1 expression (≥ 1% TPS) and driver mutations in malignant pleural fluid (A) and pleural tissues with cancer involvement (B). Figure S2. (A) Five‐year overall survival and (B) progression‐free survival in no PD‐L1 cohort with or without driver gene mutations. (C) Five‐year overall survival and (D) progression‐free survival in the high PD‐L1 cohort with a KRAS mutation or without driver gene mutations. [file CAM4-13-e70262-s002.zip › Figure S2B.jpg]

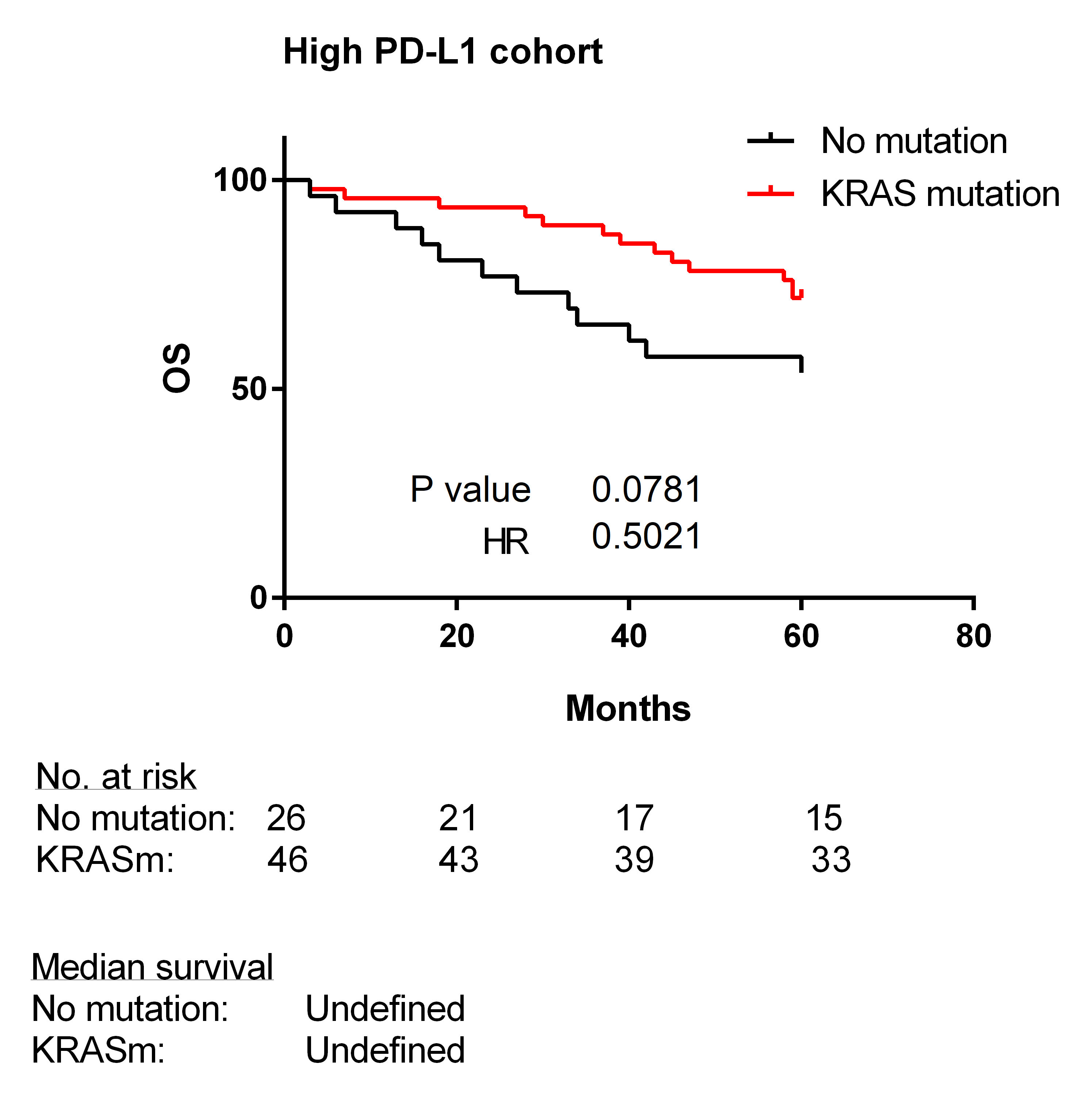

Supplement: Supplementary file 1 — Figure S1. Status of PD‐L1 expression (≥ 1% TPS) and driver mutations in malignant pleural fluid (A) and pleural tissues with cancer involvement (B). Figure S2. (A) Five‐year overall survival and (B) progression‐free survival in no PD‐L1 cohort with or without driver gene mutations. (C) Five‐year overall survival and (D) progression‐free survival in the high PD‐L1 cohort with a KRAS mutation or without driver gene mutations. [file CAM4-13-e70262-s002.zip › Figure S2C.jpg]

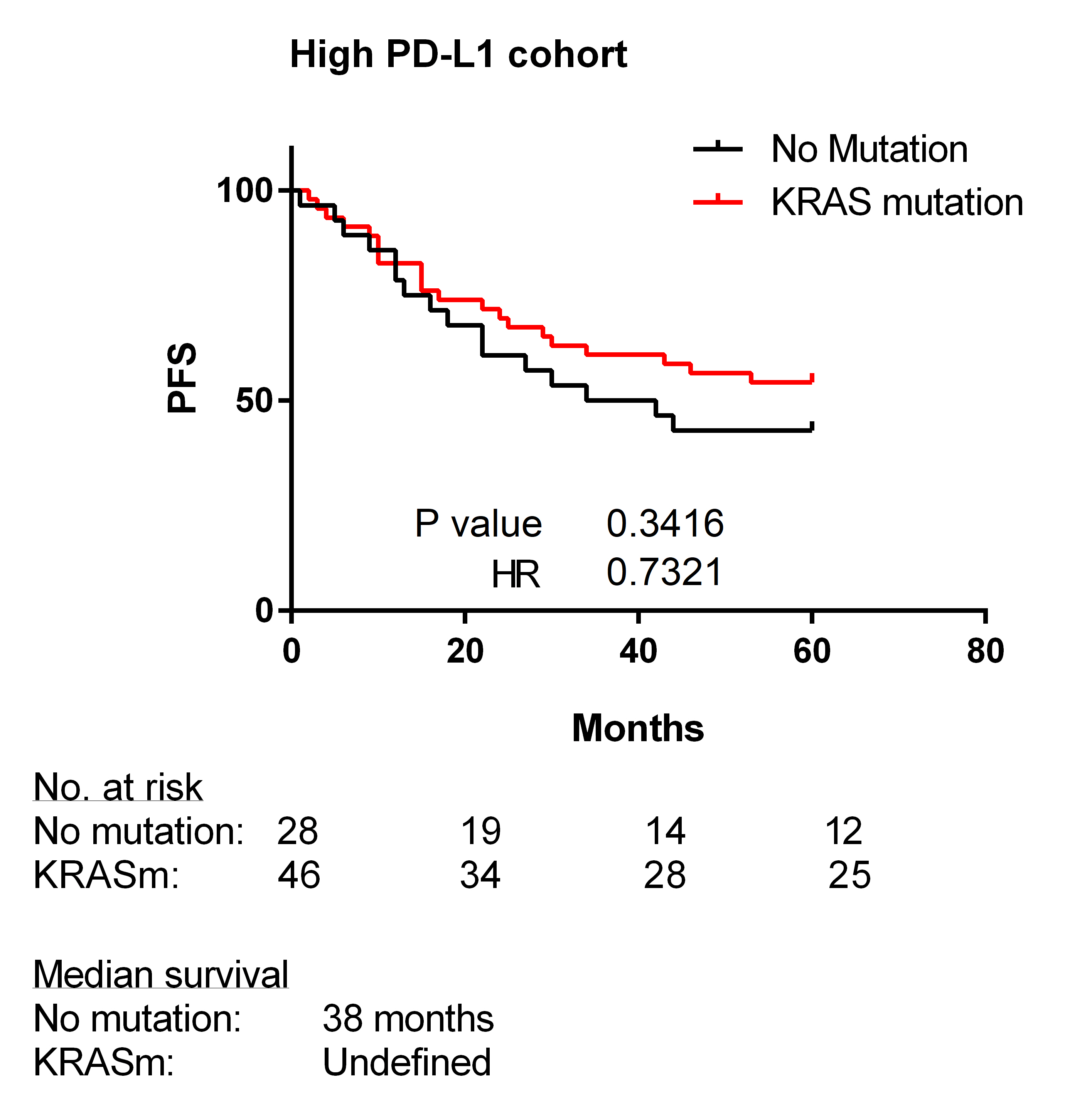

Supplement: Supplementary file 1 — Figure S1. Status of PD‐L1 expression (≥ 1% TPS) and driver mutations in malignant pleural fluid (A) and pleural tissues with cancer involvement (B). Figure S2. (A) Five‐year overall survival and (B) progression‐free survival in no PD‐L1 cohort with or without driver gene mutations. (C) Five‐year overall survival and (D) progression‐free survival in the high PD‐L1 cohort with a KRAS mutation or without driver gene mutations. [file CAM4-13-e70262-s002.zip › Figure S2D.jpg]
