# Supplementary material for: Temporal Effect on PD‐L1 Detection and Novel Insights Into Its Clinical Implications in Non–Small Cell Lung Cancer
Source: Cancer Med. 2024 Oct 9;13(19):e70262. doi: 10.1002/cam4.70262 (PMC11462595; doi:10.1002/cam4.70262)
Supplement: Supplementary file 2 — Table S1. Relationship of gender status and smoking history on PD‐L1 expression (PD‐L1 assessed from fresh tissues). Table S2. PD‐L1 expression and disease status in NSCLC cases from the tumor bank cohort (PDL1 assessed from fresh tissues). Table S3. PD‐L1 and driver gene mutation in metastatic lymph nodes and primary tumors with and without lymph node involvement. [file CAM4-13-e70262-s001.docx]

**Supplementary Table 1. Relation of gender status and smoking history on PD-L1 expression (PD-L1 assessed from fresh tissues)**

| **Characteristics** | **Gender (n, %)** | | **P value** |
| --- | --- | --- | --- |
| **Gender and PD-L1** |  |  |  |
| **TPS** | **Female** | **Male** |  |
| **<1%** | 771 (37.50) | 558 (36.30) | *0.513* |
| **1-49%** | 740 (35.99) | 582 (37.87) |  |
| **≥ 50%** | 545 (26.51) | 397 (25.83) |  |
| **Smoking history and PD-L1** |  |  |  |
| **TPS** | **Never smoked** | **Smoked** |  |
| **<1%** | 38 (54.29) | 208 (35.49) | *0.003* |
| **1-49%** | 24 (34.29) | 223 (38.05) |  |
| **≥ 50%** | 8 (11.43) | 155 (26.45) |  |

**Supplementary Table 2. PD-L1 expression and disease status in NSCLC cases from tumor bank cohort (PDL1 assessed from fresh tissues)**

| **Characteristics** | **No of cases (%)** | | | **P value** |
| --- | --- | --- | --- | --- |
| **Tumor stage** | **I** | **II** | **III** |  |
| **TPS** |  |  |  |  |
| **<1%** | 178 (42.69) | 38 (30.89) | 21 (24.42) | *0.0001* |
| **1-49%** | 173 (41.49) | 44 (35.77) | 30 (34.88) |  |
| **≥ 50%** | 86 (20.62) | 41 (33.33) | 35 (40.70) |  |

**Supplementary Table 3. PD-L1 and driver gene mutation in metastatic lymph nodes and primary tumors with and without lymph node involvement**

| **Characteristics** | **No. of cases (%)** | |
| --- | --- | --- |
| **PD-L1 Score** |  |  |
| Metastatic lymph nodes originated from lung | All histological types (n= 124) | Adenocarcinoma (n= 97) |
| TPS <1% | 28 (22.58) | 22 (22.68) |
| TPS 1-49% | 39 (31.45) | 27 (27.84) |
| TPS ≥ 50% | 57 (45.97) | 48 (49.48) |
| Primary tumors with lymph node involvement | All histological types (n=129) | Adenocarcinoma (n= 90) |
| TPS <1% | 34 (26.36) | 21 (23.33) |
| TPS 1-49% | 42 (32.56) | 30 (33.33) |
| TPS ≥ 50% | 53 (41.09) | 39 (43.33) |
| Primary tumors without lymph node involvement | All histological types (n= 498) | Adenocarcinoma (n= 367) |
| TPS <1% | 200 (40.16) | 149 (40.60) |
| TPS 1-49% | 197 (39.56) | 153 (41.69) |
| TPS ≥ 50% | 101 (20.28) | 65 (17.71) |
| **PD-L1 and driver gene mutation status** |  |  |
| Metastatic lymph nodes originated from lung | All histological types (n= 108) | Adenocarcinoma (n= 89) |
| <1% TPS /No mutation | 9 (8.33) | 7 (7.87) |
| >1% TPS/Mutation | 14 (12.96) | 13 (14.61) |
| >1% TPS/No mutation | 30 (27.78) | 21 (23.60) |
| >1% TPS/Mutation | 55 (50.93) | 48 (53.93) |
| Primary tumors with lymph node involvement | All histological types (n= 129) | Adenocarcinoma (n= 90) |
| <1% TPS /No mutation | 18 (13.95) | 6 (6.67) |
| >1% TPS/Mutation | 16 (12.40) | 15 (16.67) |
| >1% TPS/No mutation | 30 (23.26) | 15 (16.67) |
| >1% TPS/Mutation | 65 (50.39) | 54 (60.0) |
| Primary tumors without lymph node involvement | All histological types (n= 498) | Adenocarcinoma (n= 367) |
| <1% TPS /No mutation | 104 (20.88) | 60 (16.35) |
| >1% TPS/Mutation | 96 (19.28) | 89 (24.25) |
| >1% TPS/No mutation | 138 (27.71) | 76 (20.71) |
| >1% TPS/Mutation | 160 (32.13) | 142 (38.69) |
